# When Scientific Reassessment Fails Citizen Science. Comment on Soldo, A. When Citizen Science Becomes Speculation: Evaluating the Reliability of Lamnid Shark Identification from Photographic Records in the Mediterranean. *J. Mar. Sci. Eng*. 2026, *14*, 173

**DOI:** 10.3390/jmse14131148

**Published:** 2026-06-23

**Authors:** Patrick L. Jambura, Julia Türtscher, Joshua K. Moyer, Kenshu Shimada, Ilija Ćetković, Branko Dragičević, Pero Ugarković, Mišo Pavičić, Phillip C. Sternes, Simone Niedermüller, Jacopo Bernardi, Matteo Barbato, Frederik H. Mollen, Jürgen Kriwet, Douglas J. Long, Taketeru Tomita, Charlie J. Underwood, Clinton Duffy, Craig P. O’Connell, Alessandro De Maddalena, John Chisholm, Gregory B. Skomal

**Affiliations:** 1 Department of Palaeontology, University of Vienna, 1090 Vienna, Austria; 2 The MECO Project, 546 45 Thessaloniki, Greece; 3 Johannes Kepler University Linz Library, Altenberger Straße 69, 4040 Linz, Austria; 4 Department of Ecology and Evolutionary Biology, Yale University, New Haven, CT 06511, USA; 5 Peabody Museum of Natural History, Yale University, New Haven, CT 06511, USA; 6 Department of Environmental Science and Studies, DePaul University, Chicago, IL 60614, USA; 7 Department of Biological Sciences, DePaul University, Chicago, IL 60614, USA; 8 Sternberg Museum of Natural History, Fort Hays State University, Hays, KS 67601, USA; 9 Field Museum of Natural History, Chicago, IL 60605, USA; 10 Institute of Marine Biology, University of Montenegro, 85330 Kotor, Montenegro; 11 Institute of Oceanography and Fisheries, Laboratory of Ichthyology and Coastal Fisheries, 21000 Split, Croatia; 12 Independent Researcher, 21000 Split, Croatia; 13 Education and Conservation Department, SeaWorld, San Diego, CA 92109, USA; 14 Department of Biology, San Bernardino Valley College, San Bernardino, CA 92410, USA; 15 Shark Measurements, London, UK; 16 World Wide Fund for Nature Mediterranean Marine Initiative (WWF MMI), 00161 Rome, Italy; 17 Experimental Center for Habitats Conservation (CESTHA), 48122 Marina di Ravenna, Italy; 18 Department of Biology, University of Padova, 35131 Padova, Italy; 19 Institute for Marine Biological Resources and Biotechnology (IRBIM), Italian National Research Council (CNR), 91026 Mazara del Vallo, Italy; 20 Elasmobranch Research Belgium, Rehaegenstraat 4, 2820 Bonheiden, Belgium; 21 Vienna Doctoral School of Ecology & Evolution (VDSEE), University of Vienna, 1030 Vienna, Austria; 22 Department of Ichthyology, California Academy of Sciences, San Francisco, CA 94118, USA; 23 Okinawa Churashima Research Center, Okinawa Churashima Foundation, Motobu-cho 905-0206, Okinawa, Japan; 24 Okinawa Churaumi Aquarium, Okinawa Churashima Foundation, Motobu-cho 905-0206, Okinawa, Japan; 25 School of Natural Sciences, Birkbeck College, London WC1E 7HX, UK; 26 Auckland War Memorial Museum, The Domain, Parnell, Auckland 1010, New Zealand; 27 Montauk Shark Lab, Montauk, New York, NY 11954, USA; 28 School for Marine Science and Technology, University of Massachusetts Dartmouth, New Bedford, MA 02744, USA; 29 Shark Museum, 26 Forest Hill Road, Simon’s Town, Cape Town 7975, South Africa; 30 Fisheries Science and Emerging Technologies Program, Anderson Cabot Center for Ocean Life at the New England Aquarium, Boston, MA 02110, USA; 31 Massachusetts Division of Marine Fisheries, New Bedford, MA 01950, USA

## Abstract

Soldo [1] raises concerns about the reliability of citizen science-derived photographic records for identifying lamnid sharks in the Mediterranean Sea. Using a recently published juvenile white shark from Croatian waters [2] as a representative case, the author reinterprets the specimen as a porbeagle shark (*Lamna nasus*) rather than a white shark (*Carcharodon carcharias*), based primarily on selected dental characters. Given the critically endangered status of white sharks in the Mediterranean [3] and the limited number of confirmed observations [4,5], occurrences of the species in this region serve as valuable population data points [6,7] and merit careful examination. Species identifications and reinterpretations should therefore be well-supported, which includes verification of source materials and firsthand communication with citizen scientists, and make use of multiple diagnostic characters whenever possible. Here, we re-examined the specimen using all observable external and dental characters and show that it is consistent with the white shark. We further demonstrate that reliance on single, variably expressed traits—particularly dental characters affected by ontogeny and individual variation—can lead to erroneous identifications, and emphasize that rigorous taxonomic identifications should depend on a holistic, integrative assessment of all available morphological (and if available genetic) evidence.

*Carcharodon carcharias* and *Lamna nasus* both belong to the family Lamnidae and can appear similar at first glance, particularly in general body shape. Nevertheless, multiple morphological traits enable taxonomic differentiation: (1) eyes small in *C. carcharias* vs. larger eyes (its diameter about 30% as long as snout in front of mouth) in *L. nasus* (2) posterior margin of the first dorsal fin concave in *C. carcharias* vs. straight or convex in *L. nasus*; (3) first dorsal fin without a white free rear tip in *C. carcharias* vs. conspicuous white free rear tip present in *L. nasus*; (4) distinct black markings on the ventral side of the pectoral fin tips present in *C. carcharias* vs. ventral surface of the pectoral fins exhibiting a broad dark area extending from the apex toward the base in *L. nasus*; (5) secondary caudal keel below the main keel on the caudal fin absent in *C. carcharias* vs. present in *L. nasus*; (6) upper teeth broadly triangular and labiolingually flattened in *C. carcharias* vs. upper teeth narrower and not labiolingually compressed in *L. nasus*; (7) cutting edges serrated in teeth of *C. carcharias* vs. unserrated in *L. nasus*; and (8) lateral cusplets absent in teeth of *C. carcharias* vs. present in *L. nasus* (occasionally absent in very young individuals) [8–15] (Figure 1). Ontogenetic change can affect the expression of individual characters in both species, particularly dental features [8,16–20]. However, the overall diagnostic framework remains robust, as species identification is supported by the combined concordant expression of multiple independent traits.

Soldo [1] first questions the reported total length of approximately 120–130 cm, arguing that this would represent the smallest free-swimming *C. carcharias* recorded in the Mediterranean. However, smaller free-swimming individuals (approximately 85–120 cm) have previously been documented in the Mediterranean Sea [21], and specimens of comparable size are also well documented outside this region [22,23]. In addition, documented late-term embryos reach sizes overlapping with or exceeding this range [24–26], indicating natural variation in size at birth. The reported total length therefore falls within the documented early ontogenetic range of *C. carcharias* and does not constitute evidence against this identification.

Although body size alone is acknowledged as insufficient to resolve the identification of the specimen, Soldo [1] further argues that several morphological traits are inconsistent with *C. carcharias*, focusing in particular on coloration, caudal keel development, and dental morphology. With respect to coloration, the specimen is described by Soldo [1] as having a dark bluish tone; however, the available photographs show a predominantly steel-grey overall appearance without a distinct blue hue. This coloration falls well within the documented intraspecific range of juvenile *C. carcharias*, which spans lead-grey to brownish grey or blackish tones [9,10,13,14]. Additionally, Castro ([27]: p. 284) reports that the color of *L. nasus* is “dark bluish grey to bluish brown” and notes postmortem color change. Collectively, this underscores the variability and subjectivity of coloration use as a diagnostic criterion in the absence of distinct pigmentation patterns or anatomical landmarks. In contrast, discrete pigmentation patterns can provide informative diagnostic cues. One such character concerns the ventral surface of the pectoral fins. In *C. carcharias*, the pectoral fins typically exhibit dark tips with a narrow black margin along the trailing edge, whereas in *L. nasus* the ventral surface shows a broader dark area extending from the apex toward the fin base [9,13]. The specimen illustrated by Jambura et al. [2] clearly displays dark pigmentation confined to the tip and margin of the pectoral fin, consistent with *C. carcharias* and not with *L. nasus*.

Another diagnostic pigmentation character is the white free rear tip of the first dorsal fin, which is consistently present in *L. nasus* and absent in *C. carcharias*. Although Soldo [1] argues that this feature cannot be assessed from the original images, the lateral view provided by Jambura et al. [2] in their figure 1B clearly shows the first dorsal fin, which lacks any indication of a white free rear tip, consistent with *C. carcharias* (Figure 1). In addition, the shape of the first dorsal fin exhibits a concave posterior margin, which is consistent with *C. carcharias*, but different from *L. nasus* (and other lamnids), whose posterior dorsal fin margin is either straight or convex [8,13]. A further external character discussed concerns the presence of a secondary caudal keel, which is consistently developed in *L. nasus* but absent in *C. carcharias* [9,10,13,14]. Like the first dorsal fin, Soldo [1] argued that this feature cannot be assessed from the available photographic material. However, in figure 1A of Jambura et al. [2] and figure 1 of Soldo [1], the caudal peduncle seemingly lacks a secondary keel below the main caudal keel, a condition consistent with *C. carcharias*. However, given the limited image resolution, viewing angle, and partial obscuration of the caudal region in the photographs, the presence or absence of a weakly developed secondary keel cannot be determined with complete confidence. This character is therefore treated conservatively and is not considered decisive here on its own. Importantly, even when this feature is excluded from consideration, the remaining external characters collectively support identification of the specimen as *C. carcharias*.

Despite the evidence of the external morphology strongly indicative of *C. carcharias*, Soldo [1] dismisses it and states that dental morphology represents the only character set that can be reliably used for species identification in this case. The paper places particular emphasis on the inclination of the intermediate tooth, serrations (or supposed lack thereof) on the cutting edge, and the absence of lateral cusplets. With regard to tooth inclination, it is argued that the polarity of the third anterior tooth (intermediate tooth) is inconsistent with *C. carcharias*, as it is inclined distally and not mesially and therefore resembles the state observed in *L. nasus* [1]. However, this interpretation is limited for two reasons. First, the mesially inclined third anterior tooth (=intermediate tooth) that characterizes *C. carcharias* refers to the upper jaw [9,28–30], whereas the available photographs show only the lower jaw dentition; the teeth in the upper jaw are not visible and therefore cannot be evaluated. Second, inclination of the third anterior (=intermediate) tooth of the lower jaw is variable in *C. carcharias*, and both mesial and distal orientations, as well as almost no inclination, have been reported [16,29], limiting its diagnostic utility. Moreover, variation in tooth inclination, including distally inclined upper intermediates, has been documented within *C. carcharias*, particularly among males [18], further limiting the taxonomic value of this character when considered in isolation.

The second dental character discussed concerns the development of a serrated cutting edge. It is argued that the cutting edges of the visible teeth appear largely smooth and unserrated, with only small irregular areas that are interpreted as the result of wear or minor surface irregularities rather than true serrations [1]. Based on this interpretation, Soldo [1] argues that the dentition is considered inconsistent with *C. carcharias*. However, the available images show that serrations are consistently present at the basal portion of all visible teeth, including teeth that are not fully erect and therefore unlikely to have experienced functional wear. This makes abrasion an unlikely explanation for the observed morphology. Instead, restriction of serrations to the basal part of the crown is consistent with incompletely developed serrational cutting edges, a condition known to occur in full-term embryos and juvenile *C. carcharias* [16,19,24,25,30,31] and reflects ontogenetic heterodonty rather than taxonomic distinction. Reduced or basally confined serrations, even the complete absence of such, are well known in small *C. carcharias* and typically become more fully expressed with increasing body size [9,16,30]. Consequently, the observed serration pattern falls within documented intraspecific and ontogenetic variations of *C. carcharias* and does not provide support for reassignment of the specimen to *L. nasus*.

The central pillar of the reassessment of Soldo [1] is the alleged absence of lateral cusplets, which is treated as the most decisive diagnostic character. It is argued that small or juvenile *C. carcharias* consistently possess distinct lateral cusplets, whereas young *L. nasus* frequently lack them. Hence, it is concluded that the absence of such structures as reported by Jambura et al. [2] is incompatible with the identification of the specimen as a young-of-the-year *C. carcharias*. This argument, however, rests on an imprecise and historically inconsistent use of terminology that conflates non-homologous dental structures. As demonstrated by Bemis et al. [32], the lateral projections observed in juvenile *C. carcharias* are not true lateral cusplets *sensu stricto*, as found in lamnids such as *L. nasus*, but represent structures derived from the serrated cutting edge rather than independent cusps. Imprecise terminology applied to these fundamentally different morphologies has long obscured interpretations of character origin and homology in lamnid shark dentitions, with potential consequences for phylogenetic inferences [33,34]. Importantly, “serrational cusplets” (*sensu* Bemis et al. [32]) in *C. carcharias* show pronounced ontogenetic and intra-individual variability. For example, they may form distinct cusp-like projections [16,25,30], or partially to completely merge with adjacent serrations [16,20,32,35]. Their expression can also vary between tooth files and even between adjacent teeth within a single individual as can be seen in a recent case of a juvenile *C. carcharias* from Spain [35] (also see Figure 2). In the specimen illustrated by Jambura et al. [2], true lateral cusplets are indeed absent, consistent with the original description; however, weak basal projections consistent with serrational cusplets appear to be present in some teeth, whereas in others they likely are obscured by the overlying soft tissue, a condition consistent with known variation in juvenile *C. carcharias*, but not *L. nasus*.

Dental characters in sharks are well documented to exhibit substantial inter- and intraspecific morphological variation. Such variation is not restricted to ontogenetic change [17,36–39], but also includes monognathic and dignathic [40–42], geographic [43], and gynandric heterodonty [44–46], all of which are widespread across extant shark taxa, including *C. carcharias* [16,18–20]. In addition, pathologies [47,48] and pronounced individual variation [16,18] can further limit the diagnostic value of a single dental trait observed in a lone specimen. Lateral cusplets and cusplet-like structures are potentially among the most variable features, showing substantial variation in presence, number, size, symmetry, and degree of expression both within and among species [32]. A recent study by Ng et al. [49] reported a striking single-specimen case in which different teeth within one individual of the lamniform shark *Odontaspis noronhai* exhibited a mosaic of dental characters: some teeth displayed cusplet numbers previously regarded as diagnostic for the closely related species *O. ferox*, whereas others conformed to the typical condition of *O. noronhai*. Despite this pronounced intra-individual heterogeneity, the specimen was unambiguously identified as *O. noronhai* based on concordant overall morphology and molecular evidence. Similarly, Moyer et al. [50] recently documented variation in the number and development of lateral cusplets in a specimen of *L. nasus*. Nonetheless, lateral cusplets in lamniforms are still frequently treated as rigid, diagnostically decisive characters in recent taxonomic assessments, leading to misidentifications among lamniform sharks when other dental or external morphological characters are ignored or downweighed [51–53]. Awareness of these limitations is increasingly recognized in palaeontology, where taxonomic identification frequently relies on isolated teeth and consequently necessitates explicit consideration of heterodonty, ontogeny, and individual variation [54,55]. For extant sharks, however, dental characters rarely constitute the sole available evidence, and while teeth remain highly informative, robust taxonomic identification must integrate dental data with external morphology and, where available, molecular evidence. Treating a single, variably expressed trait as diagnostically decisive is ill-advised when multiple lines of evidence are available. We therefore maintain that best practices employ a holistic approach to taxonomic identification in which seemingly aberrant traits are contextualized or further studied rather than overemphasized to the exclusion of a larger body of evidence.

The original identification of the specimen by Jambura et al. [2] was based on the evaluation of both external and dental characters. Here, we have explicitly revisited these characters with particular emphasis on their documented ontogenetic, positional, and individual variability. We also acknowledge that such variability does not weaken taxonomic inference but is a prerequisite for its correct application. When all observable characters are considered collectively and interpreted within their known limits, the specimen consistently falls within the known morphological range of *Carcharodon carcharias* and does not match the diagnostic framework of *Lamna nasus*. Genetic analyses would have provided an additional independent line of evidence, but could not be conducted by the authors because the specimen was sold following capture [2]. Nevertheless, where feasible, molecular approaches should be incorporated into future assessments to further strengthen taxonomic identifications and the scientific value of citizen science-derived observations.

Citizen science has become an increasingly important tool in shark and ray research [56–58], particularly in regions such as the Mediterranean Sea where scientific survey data remain scarce and many species are rarely encountered. Opportunistic observations provided by fishers, divers, and the public have substantially improved knowledge on species occurrence, distribution, behaviour, and seasonality, especially for elusive or threatened taxa in this area [59–64]. At the same time, citizen science-derived records require careful verification, as photographic material may be incomplete, misleading, or incorrectly interpreted. A notable example is the purported first Mediterranean record of the goblin shark *Mitsukurina owstoni* [65], which was later challenged [66] and ultimately retracted [67] after concerns were raised that the photographed specimen represented a plastic toy rather than a natural specimen. Such cases highlight that citizen science observations should not be accepted at face value, but instead require critical expert evaluation, verification of source material, and integrative assessment using all available lines of evidence. When subjected to rigorous scrutiny, however, citizen science records remain a powerful and often indispensable source of conservation-relevant biodiversity data.

Reliable identification of white sharks is of particular relevance in the Mediterranean Sea, where the species is extremely rare and direct observations remain scarce despite repeated research efforts. While recent studies using eDNA [68,69] confirmed the presence of white sharks in the region, systematic field-based surveys have so far failed to collect observational or specimen-based data [70,71]. Consequently, current knowledge of occurrence and distribution relies mainly on meta-analyses of opportunistic records [4–7], including photographic observations contributed by fishers and members of the public [2,26,72–75]. Although such data cannot replace targeted field studies, they represent an essential source of information in systems where conventional survey approaches have proven ineffective. The current case of a juvenile white shark from the Adriatic Sea illustrates that the scientific value of such records depends on careful expert evaluation grounded in a clear understanding of character variability. When assessed within an appropriate taxonomic framework, citizen science observations can therefore be a powerful source of information to document rare species occurrences and inform conservation-relevant assessments.

In this context, dismissing well-documented records without rigorous, integrative scrutiny risks undermining conservation-relevant assessments. This is particularly critical for observations of juvenile or newborn individuals, as such records may provide rare indications of potential nursery or parturition areas in a region where scientific survey data remain limited. Given the low detection rates and the highly threatened population status of white sharks in the Mediterranean [6,7], careful and methodologically rigorous assessment of all available evidence is essential. Rather than weakening scientific standards, integrative evaluation strengthens the reliability of such records and ensures that conservation-relevant information is neither overstated nor prematurely disregarded.

## Figures and Tables

**Figure 1:**
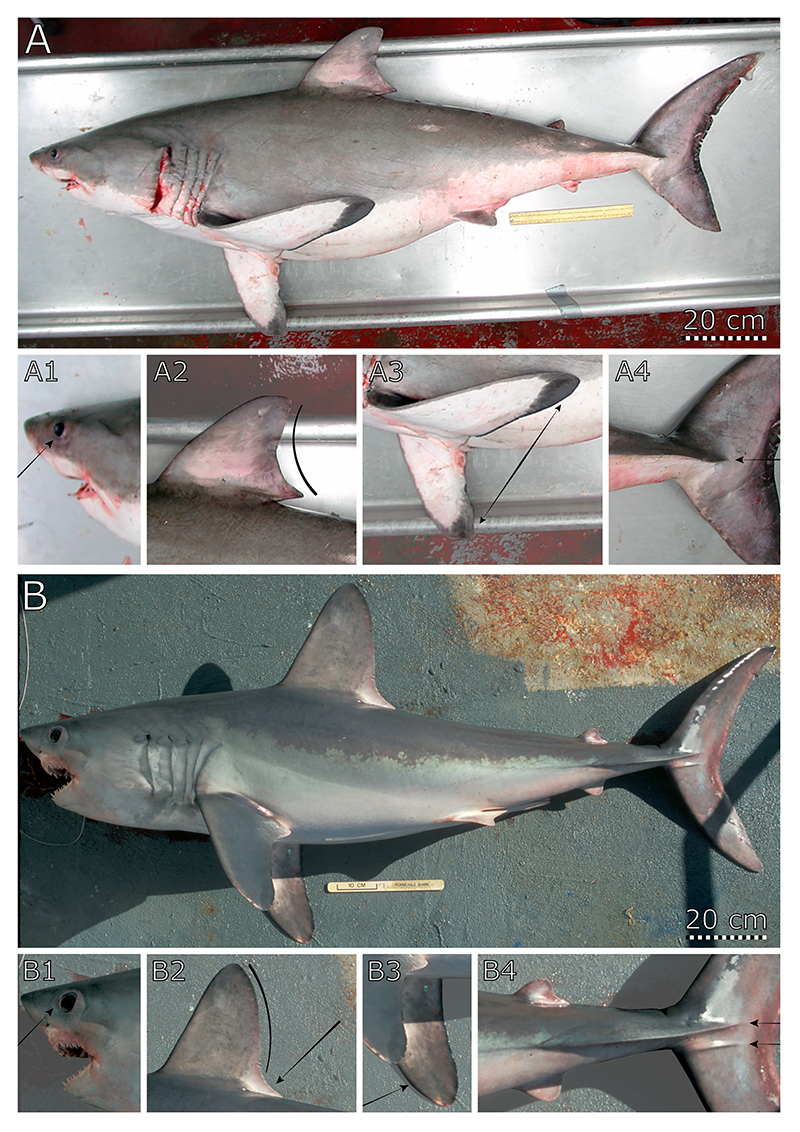
External diagnostic characters distinguishing a juvenile *Carcharodon carcharias* ((**A**); 183 cm TL) from a similarly sized *Lamna nasus* ((**B**); 188 cm TL). Diagnostic differences include relative eye size (**A1,B1**), posterior margin and pigmentation of the first dorsal fin (**A2,B2**), ventral pectoral fin markings (**A3,B3**), and caudal keel morphology (**A4,B4**).

**Figure 2:**
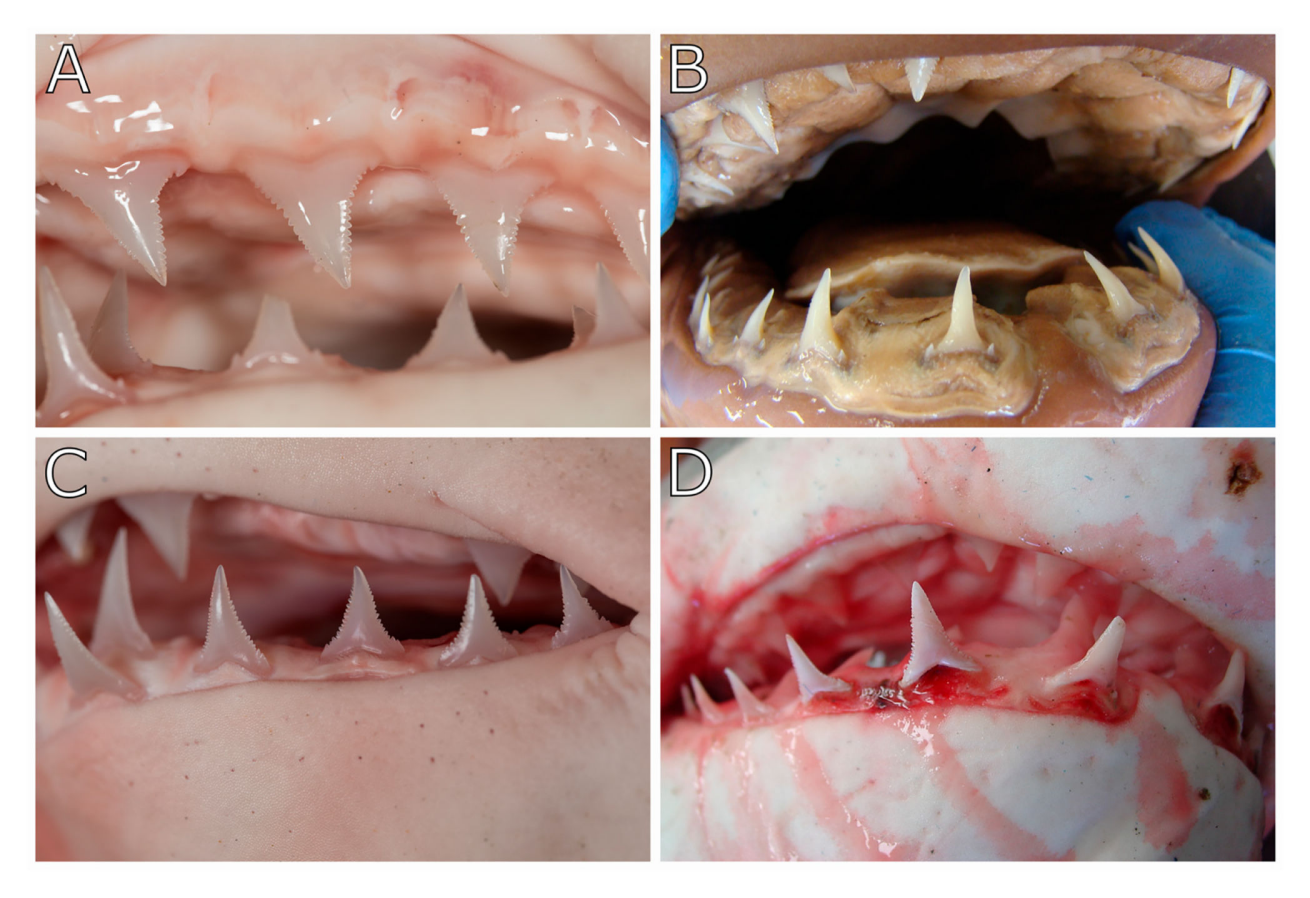
Variation in the development of serrational cusplets in juvenile white sharks (*Carcharodon carcharias*). (**A**). AIM MA122232 (159.4 cm TL); (**B**). OCF-P19920522-1 (TL: 135 cm); (**C**). Rehutai AIM MA125777 (105 cm TL); (**D**). NMFS uncatalogued (183 cm TL). Abbreviations: AIM—Auckland Museum; OCF—Okinawa Churashima Research Center; NMFS—National Marine Fisheries Service.
